# Parasitic Worms: Knowledge, Attitudes, and Practices in Western Côte d’Ivoire with Implications for Integrated Control

**DOI:** 10.1371/journal.pntd.0000910

**Published:** 2010-12-21

**Authors:** Cinthia A. Acka, Giovanna Raso, Eliézer K. N'Goran, Andres B. Tschannen, Isaac I. Bogoch, Essane Séraphin, Marcel Tanner, Brigit Obrist, Jürg Utzinger

**Affiliations:** 1 Unité de Formation et de Recherche (UFR) Sciences Sociales, Université de Cocody-Abidjan, Abidjan, Côte d'Ivoire; 2 Département Environnement et Santé, Centre Suisse de Recherches Scientifiques en Côte d'Ivoire, Abidjan, Côte d'Ivoire; 3 Department of Epidemiology and Public Health, Swiss Tropical and Public Health Institute, Basel, Switzerland; 4 University of Basel, Basel, Switzerland; 5 Unité de Formation et de Recherche (UFR) Biosciences, Université de Cocody-Abidjan, Abidjan, Côte d'Ivoire; 6 Division of Infectious Diseases, Massachusetts General Hospital, Boston, Massachusetts, United States of America; 7 Institute of Social Anthropology, University of Basel, Basel, Switzerland; The George Washington University Medical Center, United States of America

## Abstract

**Background:**

In the developing world where parasitic worm infections are pervasive, preventive chemotherapy is the key strategy for morbidity control. However, local knowledge, attitudes, and practices (KAP) of parasitic worms are poorly understood, although such information is required for prevention and sustainable control.

**Methods:**

We carried out KAP surveys in two rural communities of Côte d'Ivoire that were subjected to school-based and community-based research and control activities. We used qualitative and quantitative methods. The former included observations, in-depth interviews with key informants, and focus group discussions with school children and adults. Quantitative methods consisted of a structured questionnaire administered to household heads.

**Principal Findings:**

Access to clean water was lacking in both communities and only a quarter of the households had functioning latrines. There was a better understanding of soil-transmitted helminthiasis than intestinal schistosomiasis, but community-based rather than school-based interventions appeared to improve knowledge of schistosomiasis. In the villages with community-based interventions, three-quarters of household interviewees knew about intestinal schistosomiasis compared to 14% in the village where school-based interventions were implemented (*P*<0.001). Whereas two-thirds of respondents from the community-based intervention village indicated that the research and control project was the main source of information, only a quarter of the respondents cited the project as the main source.

**Conclusions/Significance:**

Preventive chemotherapy targeting school-aged children has limitations, as older population segments are neglected, and hence lack knowledge about how to prevent and control parasitic worm infections. Improved access to clean water and sanitation is necessary, along with health education to make a durable impact against helminth infections.

## Author Summary

There is a need to better understand communities' knowledge, attitudes, and practices (KAP) of neglected tropical diseases to improve prevention and control efforts. We studied the socio-cultural aspects of parasitic worm infections in two villages (Mélapleu and Zouatta II) of western Côte d'Ivoire, where research and control activities have been implemented. Zouatta II was exposed to a community-based approach, while school-based interventions were implemented in Mélapleu. KAP surveys were carried out using qualitative and quantitative methods. Although there was some knowledge of parasitic worm infections in both villages, we found important differences between the two villages regarding intestinal schistosomiasis: there was a better understanding of this disease in Zouatta II. However, even the community-based research and control efforts implemented in Zouatta II were ineffective in transforming the information conveyed into preventive behavior related to water contact. Our results suggest that KAP of parasitic worm infections conveyed by research and control activities targeting only school-aged children have shortcomings as older population groups are left out. Hence, for effective control of parasitic worms, children and adults must be educated and interventions should include access to deworming drugs, clean water and sanitation.

## Introduction

Parasitic worms (helminths), such as soil-transmitted helminths and schistosomes, were so common in the 1940s that Norman W. Stoll coined the term “this wormy world” [1]. Helminths continue to affect hundreds of millions of people today. Indeed, more than half of the human population is at risk of soil-transmitted helminthiasis and schistosomiasis with more than 1 billion people infected, possibly causing a global burden of more than 40 million disability-adjusted life years (DALYs) lost annually [2]–[7]. Improvements have been made to reduce helminth transmission in many parts of the world [8], but worm infections continue to be an issue of major public health and socio-economic concern. Helmintic diseases disproportionately affect those in poverty [9], [10], with the poorest of the poor commonly suffering from multiple species infections concurrently [11]–[13].

In 2001 a resolution was passed during the 54^th^ World Health Assembly (WHA) with the target (for member states) to regularly administer anthelmintic drugs to at least 75% and up to 100% of all school-aged children at risk of morbidity due to schistosomiasis and soil-transmitted helminthiasis by 2010 [14]. In the same year, the World Health Organization (WHO) assembled an expert committee to refine the global strategy for the prevention and control of schistosomiasis and soil-transmitted helminthiasis. Since then, millions of school-aged children have received anthelmintic drugs (albendazole or mebendazole against soil-transmitted helminthiasis and praziquantel against schistosomiasis) [15]–[17]. Comparatively little emphasis has been put on improving access to clean water and adequate sanitation and changing hygiene behavior, although these interventions represent an effective way for preventing intestinal parasitic infections, and are key factors for sustainable control [17]–[20].

Health education and promotion campaigns are essential for any change in behavior to be made. For health education and promotion activities to be effective, target audiences must be identified so that a clear message can be delivered, and hence local knowledge and perceptions must be taken into account [21]. Recent studies support that both individual and community perceptions and attitudes of parasitic worm infections and their prevention and treatment are important factors [22]–[24].

Over the past 15 years, we carried out research and control activities against schistosomiasis, soil-transmitted helminthiasis, and malaria in the region of Man in western Côte d'Ivoire, including studies which assessed local perceptions toward parasitic worm infection, drug intervention studies, risk mapping and prediction, and community-based control activities [25]–[32]. Here we describe knowledge, attitudes, practices (KAP) and behavior related to helminth infections in two rural communities of western Côte d'Ivoire that were subjected to either school-based or community-based research and control activities. The experience gained from this study might be useful for the design and implementation of an integrated control program and the lessons learned might stimulate other control programs to think and act beyond preventive chemotherapy.

## Methods

### Study area and historical context

The Man region in western Côte d'Ivoire is populated by four main ethnic groups: Guéré, Toura, Wobé, and Yacouba. This study was conducted in the villages of Mélapleu (primarily Yacouba) and Zouatta II (primarily Wobé) between October 2003 and June 2004. Details of the study villages, with particular emphasis on intestinal helminth infections, have been described elsewhere [11], [25], [33]. At the time of the survey there were no health facilities available in either village. The health center nearest to Mélapleu was situated in the neighboring village of Gbatongouin, some 5 km east. With regard to Zouatta II, the nearest health center was located in Facobly, approximately 10 km away. In the district town of Man, some 25 km away from each village, there is a hospital. Of note, due to an armed conflict and socio-political unrest that occurred in Côte d'Ivoire since September 2002 [30], only the hospital of Man was functional at the time of the study and was run by the non-governmental organization ‘Médecins sans Frontiers’, providing free medical care for those in need. Apart the research activities undertaken by the Centre Suisse de Recherches Scientifiques en Côte d'Ivoire (CSRS), in partnership with the Université de Cocody-Abidjan and the Swiss Tropical and Public Health Institute (Swiss TPH), only very limited actions against schistosomiasis, soil-transmitted helminthiasis and malaria had taken place in this area.

A pilot study was carried out in Mélapleu and two neighboring villages in 1996 to develop a questionnaire for the rapid identification of school children at risk of intestinal schistosomiasis [25]. Self-reported blood in stool proved a promising symptom, and hence the questionnaire was administered at the regional level, first in 1997 and again in 2002. Questionnaire results were validated with parasitological results obtained from 57–60 randomly selected schools using Kato-Katz thick smear examinations of stool samples from up to 100 children per school [26], [28]. The parasitological data were subsequently used for mapping and predicting the distribution of *Schistosoma mansoni*, hookworm, and *S. mansoni*-hookworm co-infections [29], [34]–[37]. In addition, a study was carried out in school children to assess the day-to-day and intra-specimen variation of *S. mansoni* egg output before and after praziquantel administration [34].

In Zouatta II, a multidisciplinary community-based study was implemented in 2002, consisting of a KAP survey pertaining to hygienic habits, water-contact and health-seeking behavior and in-depth appraisal of *S. mansoni*, soil-transmitted helminths, intestinal protozoa, and *Plasmodium* infections and multiparasitism among all family members living in 75 randomly selected households (more than half of the village) [11], [33], [38]. Infected individuals were treated and awareness was raised about how to prevent parasitic worm infections in the future with a small, local public health campaign at the time of the study.

To better understand the perception and practices of parasitic worms, we used a combination of qualitative and quantitative methods. Data were then triangulated to verify and complete information gathered from communities. While the quantitative approach permitted us to obtain information on the frequency of people reporting signs and symptoms, causes, treatment and prevention of helmintic diseases, the qualitative data allowed us to further deepen the meaning of the diseases and to assess actual practice.

### Qualitative methods

For the current study, a two-step qualitative approach was adopted. First, observations were made over a 2-week period at community places such as churches, local markets, schools, and water collection and washing sites. This enabled us to identify potential key informants and water contact sites for the second phase of the study. In addition, we observed and collected information on means of communication between formal and informal groups – such as youth, women, congregations, community assistance groups, and community leaders (e.g. meetings within and between groups, radio, and television). Finally, 30 focus group discussions (FGDs) and five semi-structured interviews were conducted.

The FGDs (20 in Zouatta II, 10 in Mélapleu) were conducted with elderly women, adult women, adult men and school children. Common diseases, knowledge of signs and symptoms, causes, treatment, and prevention of schistosomiasis and intestinal helminths, as well as water use and sanitation practices were discussed. The semi-structured interviews were conducted with traditional healers (three in Zouatta II, two in Mélapleu). The roles of traditional healers in the community and common diseases they deal with were discussed. The traditional healers' knowledge of intestinal helminth infections and schistosomiasis was also explored. FGDs and interviews were tape-recorded and transcribed. The information was used to create a detailed reconstruction of villagers' KAP pertaining to intestinal schistosomiasis and soil-transmitted helminthiasis.

### Quantitative methods

Quantitative techniques consisted of household surveys that utilized a structured questionnaire with both closed and open-ended questions. Closed questions pertained to demographic and socio-economic factors, knowledge of local terms for schistosomiasis, and measures for preventing parasitic worm infection. Other aspects of knowledge and practices were explored by open-ended questions.

A complete list of households in each village was obtained by census and all households were enrolled. Only household heads were interviewed; if a household head was absent, an adult household member was interviewed instead. Questionnaires were administered in the local language by trained enumerators. The survey collected demographic data and assessed interviewee's knowledge and practices of parasitic worms. Because there is no local translation for intestinal schistosomiasis, we inquired about related symptoms, such as blood in stool and abdominal pain [25], [39]. Respondents were asked whether they consider intestinal worms and schistosomiasis harmful, how an individual becomes infected, type of treatment sought, how to avoid worm infections, and the source of information.

### Socio-economic status

A household-based asset approach was used to estimate the socio-economic status of each household, including principal component analysis [40]. The first principal component (PC) of asset ownership across households explained 20% of the variability and gave greatest weight to households possessing a radio (0.47), followed by a ventilator (0.42), and a television (0.42). After standardization of these weighted asset variables, households possessing a ventilator had the greatest score (1.57), followed by households with a refrigerator (1.53). The lowest scores were associated to households that lacked a bicycle (−0.47) or a radio (−0.38). The asset scores were summed to a total score attached to each household, and households were ranked according to their total score. Thereafter, households were grouped into wealth quintiles: most poor, very poor, poor, less poor, and least poor [28], [41].

### Data management and analysis

Qualitative data analysis was based on the model of systems of signs, meaning, and action [42]. It aims to identify the system of signs, meaning, and actions that contribute to individuals' perceptions, interpretations, and behavior of health. A preliminary analysis was performed on the public perception of helminth infection and then created a “code list”. This code list documents information categories, types, and contents related to public perceptions of a helminth infection. With the help of this code list, interviews and FGDs were broken down into multiple units. Coded interviews and FGDs were then entered into a Maxqda database (VERBI Software Consult, Social Research, Gmbh; Berlin, Germany). The coded data were analyzed for the frequency at which information and content categories occur, including correlations between these variables. The software also allows for the rapid retrieval of text related to one or more information categories and the identification of informants. After coding, the first level of analysis was conducted to identify different types of symptoms, perceived causes, preventative strategies, and treatment options that were outlined by respondents. Water contact behavior and sources of information were identified in the frame of the first level of analysis. The second level of analysis examined the contents of several categories of information by investigating the implied meaning associated with each category. The analysis was also conducted on the articulations and discontinuities among the system of signs, meaning, and actions.

Quantitative data were entered into EpiInfo version 6.04 (Center for Disease Control and Prevention; Atlanta, GA, USA). Analyses were performed with STATA version 8.0 (Stata Corporation; College Station, TX, USA). A chi-square (χ^2^) test was used to determine inter-village differences. χ^2^ and Fisher's exact tests were used to assess the association between KAP, demographic factors, and socio-economic variables.

### Ethical statement

The study protocol was approved by the institutional research commissions of the CSRS in Abidjan, Côte d'Ivoire and the Swiss TPH in Basel, Switzerland. The study was cleared by the Ministry of Higher Education and Research of Côte d'Ivoire, and the district health and education authorities of Man. The village chiefs in Zouatta II and Mélapleu were asked for permission to work in the village, and then a meeting was organized with the village authorities to explain the aims and procedures of the study. The purpose and activities of the study were explained to the local community including parents and teachers. Sufficient time was given to ask question and it was emphasized that participation was voluntary. Oral consent was obtained, since the majority of the participants were illiterate. A local witness was present during oral consent. Hence, we followed common procedures for studies carried out in Côte d'Ivoire when only questionnaire data are collected and no concurrent biological samples.

## Results

### Characteristics of the study population

A total of 207 interviews were conducted with household heads; 111 (53.6%) in Mélapleu and 96 (46.4%) in Zouatta II. Table 1 shows the respondents' characteristics. The two communities showed no difference with regard to age, sex, education, and occupation. However, there was a significant difference in religion; the majority of interviewees in Mélapleu were animists, whereas Christians were predominant in Zouatta II. Moreover, marital status differed significantly between the two villages with a higher proportion of married or widowed household heads in Mélapleu than in Zouatta II.

**Table 1:** Characteristics of the study populations in western Côte d'Ivoire, subjected to KAP surveys for parasitic worms in 2003/2004.

Characteristics	Total	Village		χ^2^	*P*-value
	(n = 207)^a^	Zouatta II(n = 96)a,b	Mélapleu(n = 111)a,c		
Sex					
Male	57	58	55		
Female	44	42	45	0.23	0.625
Age group (years)					
18–35	34	29	38		
36–60	44	49	39		
>60	23	22	55	2.43	0.296
Ethnicity					
Yacouba	53	3.1	96		
Wobé	40	87	0		
Others	7.2	10	4.5	N/A	<0.001^d^
Religion					
Animists	50	28	69		
Christians	32	56	11		
Moslems	13	13	14		
Atheists	4.8	3.1	6.3	N/A	<0.001^d^
Education					
Illiterate	53	54	52		
Primary school level	31	29	33		
Secondary school level or higher	16	17	14	0.48	0.783
Matrimonial status					
Married	76	73	79		
Single	6.8	13	1.8		
Divorced	2.9	5.2	0.9		
Widower	14	9.4	18	15.0	0.002
Occupation					
Farmer	92	93	91		
Fisherman	3.4	4.2	2.7		
Trader	2.9	1.0	4.5		
Teacher	0.5	1.0	0		
None	0.5	0	0.9		
Others	1.0	1.0	0.9	N/A	0.479^d^
Household assets					
Electricity	68	51	82	22.5	<0.001
Latrine	25	29	22	1.55	0.212
Well	15	27	5	20.6	<0.001
Radio	47	46	49	0.16	0.686
Television	21	13	29	8.20	0.004
Video	1.4	0	2.7	N/A	0.105^d^
Ventilator	6.8	6.3	7.2	0.07	0.784
Refrigerator	3.9	3.1	4.5	0.26	0.608
Bicycle	3.9	5.2	2.7	N/A	0.351^d^
Land possession (m^2^)					
<2000	44	55	37		
≥2000	40	15	56	47.36	<0.001

N/A, not applicable.

a All values are percentages.

b Village subjected to community-based research and control activities.

c Village subjected to school-based research and control activities.

d *P*-value based on Fisher's exact test.

### Commonly perceived diseases

The most commonly perceived disease in both villages was malaria (87%). Intestinal worms, dysentery, schistosomiasis, and scabies were reported by 52%, 50%, 29% and 22%, respectively. Schistosomiasis was perceived as a common disease by 57% respondents in Zouatta II, but only 5% reported schistosomiasis in Mélapleu (χ^2^ = 69.7, degree of freedom (d.f.) = 3, *P*<0.001). Investigators explained the term “bilharziose” (schistosomiasis in French), and by describing symptoms of the disease, as there is no local name.

The most frequently mentioned signs and symptoms in both villages were cough (74%), headache (73%), fatigue (73%), abdominal pain (72%), fever (72%), backache (71%), and diarrhea (59%).

### Perception, treatment, and prevention of intestinal worms

Local names of intestinal worms were *sion mlein* and *mlein mlein* in Wobé, and *gbihienein* in Yacouba. All household heads considered intestinal worms to be a serious disease as revealed by quantitative questionnaire results. Common beliefs were that intestinal worms cause fatigue, liver damage, anemia, and other illnesses. Signs and symptoms of intestinal worm infections (i.e., loss of appetite, worms in stool, diarrhea, and blood in stool) were more often listed by respondents in Zouatta II than in Mélapleu (Table 2). A quarter of respondents in both villages reported that at least one of their children was currently affected with, or has suffered from, intestinal worm infections in the past 6 months.

**Table 2:** Knowledge of, and measures against, intestinal worms in two villages of western Côte d'Ivoire in 2003/2004.

Variable	Total	Village		χ^2^	*P*-value
	(n = 207)	Zouatta IIa,b(n = 96)	Mélapleua,c(n = 111)		
Signs and symptoms					
Swollen stomach	93	95	91	1.10	0.293
Vomiting/nausea	92	91	93	0.32	0.571
Loss of weigh	81	80	81	0.02	0.874
Loss of appetite	74	84	65	10.2	0.001
Worms in stool	71	78	63	6.42	0.011
Diarrhea	55	66	46	8.05	0.005
Blood in stool	40	58	25	23.4	<0.001
Dysentery	16	21	12	3.19	0.074
Perceived causes					
Consumption of meat	85	84	85	<0.01	0.951
Consumption of sweets	84	84	84	0.01	0.951
Consumption of overripe fruits	83	90	77	6.01	0.014
Consumption of spoilt meal	54	65	44	8.64	0.003
Lack of hygiene	51	69	35	23.3	<0.001
Inborn disease	20	25	15	3.03	0.231
Unknown	2.4	1.0	4.0	1.42	0.231
Place of treatment					
Dispensary/hospital	69	64	73	2.12	0.145
Door-to-door sellers	64	50	76	14.7	<0.001
Hospital	61	86	40	47.6	<0.001
Family	50	64	39	12.7	<0.001
Pharmacy	38	40	37	0.15	0.696
Traditional healer	23	28	19	2.44	0.118
Type of treatment					
Pharmaceutical medicine	84	86	82	0.76	0.380
Drugs sold on street markets	58	69	49	8.53	0.003
Traditional medicine	49	57	42	4.60	0.032
Prevention (n = 152^d^)					
Washing hands before eating	81	95	64	23.3	<0.001
Avoiding overripe fruits consumption	79	89	67	10.9	0.001
Washing hands after defecation	72	84	59	12.4	<0.001
Avoiding meat consumption	71	83	57	12.2	<0.001
Avoiding sweets consumption	70	78	60	5.82	0.016
Washing fruits before consumption	65	83	44	24.8	<0.001
Wearing shoes	55	74	31	28.1	<0.001

a All values are percentages.

b Village subjected to community-based research and control activities.

c Village subjected to school-based research and control activities.

d Only people who gave an affirmative response for prevention were included in this analysis.

The semi-structured interviews and FGDs revealed that participants in both villages were familiar with intestinal helminths and that they possess knowledge about the associated illness. *Sion mlein* and *gbihienein* were perceived to have two categories of symptoms. One category includes skin alterations such as itching, swelling, and rashes. The second category includes systemic type of illnesses consisting of weight loss, abdominal bloating, loss of appetite, weakness, vomiting, and bloody stools. Each category combined different elements from the context, values, and cultural beliefs that formed the population's concept of disease severity and causality. For example, mothers knew that skin disorders were related to disease onset and, in their view, the discomfort caused by such skin disorders justified seeking medical attention, but was not an indication that the disease was serious. The common perception was that both traditional and modern medicines would stop disease progression.

The second category of symptoms was more widely perceived to be caused by worms. For example, abdominal bloating was thought to be commonly caused by worms, and the associated symptoms of fatigue, anorexia, and anemia was thought to ultimately lead to the progressive destruction of the body. Most participants considered worms as a very serious problem in children, capable of causing fatalities in the absence of early treatment.

In both villages, it was commonly believed that consumption of meat and sweetened foods were the main sources of infection. Significant differences between the two villages were noted with regard to consumption of overripe fruits (90% in Zouatta II *vs.* 77% in Mélapleu) and poor hygiene (69% in Zouatta II *vs.* 35% in Mélapleu). One-fifth of the respondents perceived intestinal worms as inborn diseases. FGDs revealed that participants did not establish a link between intestinal worm infections and contact with soil. A few women attributed worm infections in children to the habit of soil consumption as illustrated by an adult woman from Mélapleu: “*The one to two-year-old children contract worm infections from eating soil*”.

With regard to treatment, the majority of participants ranked medical treatment as the most effective approach and 84% of the household claimed to have taken anthelmintic drugs, among which 58% had taken medicine sold on local street markets and 49% traditional medicine. Medical treatment was considered to be relatively inaccessible, and hence traditional medicine and drugs from local street markets were used instead.

The importance of washing hands, washing fruits, and wearing shoes as preventive measures against intestinal worms were significantly more often reported in Zouatta II than in Mélapleu.

Despite this knowledge, most households used unprotected surface water from rivers and ponds as source of drinking water. Collection of water from wells for drinking was rare in both villages (9.5% in Zouatta II, 1.9% in Mélapleu). Functioning latrines were available in only 52 households (29% in Zouatta II, 21% in Mélapleu). However, the presence of a latrine did not guarantee its usage. The qualitative analysis revealed that open defecation (“in the bush”, in close proximity to or inside a river) was common in both communities. Young children were allowed to defecate anywhere within the compound or at the entrance of the village.

Table 3 demonstrates that there was no significant difference regarding the perceived causes of intestinal worm infection and educational attainment or religion. Interviewees who completed secondary school and Christians commonly attributed worms to a lack of food hygiene, while the illiterate individuals and animists mainly considered meat consumption as a common etiology of worm infection. There were no significant differences in the knowledge of symptoms and causes of intestinal worm infection with regard to socio-economic status (Table 4).

**Table 3:** Perceived causes, treatment, and prevention of intestinal worms, stratified by educational attainment and religion, in 2003/2004.

Variable	Total(n = 207)^a^	Educational attainment			χ^2^	*P-*value	Religion					χ^2^	*P-*value
		Illiterate(n = 110)^a^	**Primary school** **(n = 65)^a^**	**Secondary school and higher** **(n = 32)^a^**			Christians(n = 66)^a^	Moslems(n = 27)^a^	Animists(n = 104)^a^	Atheists(n = 10)^a^			
Perceived causes													
Consumption of meat	85	86	81	84	0.72	0.695	83	78	88	80	1.87		0.599
Consumption of sweets	84	85	85	78	1.01	0.602	85	85	84	80	0.19		0.979
Consumption of overripe fruits	83	85	82	78	0.78	0.675	88	78	81	80	2.00		0.571
Consumption of spoilt meal	54	58	37	72	12.5	0.002	62	44	50	60	3.54		0.315
Lack of hygiene	51	50	45	61	3.83	0.147	65	22	50	40	14.8		0.002
Inborn disease	20	25	9.0	25	6.67	0.036	24	15	19	10	0.66		0.600
Type of treatment													
Pharmaceutical medicine	48	82	89	81	1.89	0.387	91	78	84	60	7.43		0.059
Drugs sold on street markets	58	59	63	44	3.40	0.182	67	56	52	70	4.26		0.234
Traditional medicine	49	57	38	44	6.24	0.044	55	37	84	50	2.35		0.502
Prevention^b^ (n = 152)													
Washing hands before eating	81	82	76	88	1.76	0.415	86	78	75	100	4.32		0.229
Avoiding overripe fruits consumption	79	82	72	84	2.31	0.314	86	67	78	63	4.64		0.200
Washing hands after defecation	72	89	63	88	5.53	0.063	86	50	67	75	10.9		0.012
Avoiding meat consumption	71	75	69	64	1.42	0.490	81	61	68	50	0.11		0.141
Avoiding sweets consumption	70	68	76	60	2.15	0.340	81	72	65	25	N/A		0.009^c^
Washing fruits before consumption	65	67	56	80	4.74	0.093	74	50	59	88	6.40		0.094
Wearing shoes	55	59	46	60	2.34	0.310	75	39	44	38	N/A		0.001^c^

Data from the two study villages (Mélapleu and Zouatta II) in western Côte d'Ivoire were pooled.

N/A, not applicable.

a All values are percentages.

b Only people who gave an affirmative response for prevention were included in this analysis.

c *P*-value based on Fisher's exact test.

**Table 4:** Knowledge of signs and symptoms, perceived causes, treatment, and prevention of intestinal helminths, stratified by wealth quintiles, in 2003/2004.

Variable	Total(n = 207)^a^	Wealth quintile					Ratio(most poor/least poor)
		Most poor(n = 42)^a^	Very poor(n = 42)^a^	Poor(n = 41)^a^	Less poor(n = 41)^a^	Least poor(n = 41)^a^	
Signs and symptoms							
Swollen stomach	93	86	95	95	95	93	0.92
Vomiting/nausea	92	90	95	90	88	95	0.95
Loss of appetite	74	81	86	68	68	66	1.18
Loss of weight	81	71	90	76	83	83	0.86
Worms in stool	71	67	74	73	73	66	1.01
Diarrhea	55	50	52	54	59	61	0.82
Blood in stool	40	40	43	49	27	44	0.92
Dysentery	16	17	17	17	4.8	24	0.68
Perceived causes							
Consumption of meat	85	88	88	73	88	85	1.03
Consumption of sweets	84	88	81	85	85	80	1.09
Consumption of overripe fruits	83	83	83	88	80	80	1.06
Consumption of spoilt meal	54	50	57	59	49	54	0.93
Lack of hygiene	51	62	55	44	41	51	1.20
Inborn disease	20	14	17	22	19	27	0.52
Place of treatment							
Dispensary/hospital	69	60	64	66	78	76	0.78
Door-to-door sellers	64	50	67	68	68	66	0.75
Family	50	40	64	44	49	54	0.75
Type of treatment							
Pharmaceutical medicine	84	90	81	83	80	85	1.05
Drugs sold on street markets	58	48	67	59	59	59	0.81
Traditional medicine	49	40	60	41	51	54	0.75
Prevention							
Washing hands before eating	81	88	90	77	63	86	1.01
Avoiding overripe fruits consumption	79	76	90	90	77	62	1.21
Washing hands after defecation	72	79	83	61	57	83	0.95
Avoiding meat consumption	71	76	76	77	63	62	1.21
Avoiding sweets consumption	70	85	62	74	63	62	1.36
Washing fruits before consumption	65	67	83	61	60	55	1.20
Wearing shoes	55	61	76	55	37	45	1.35
Unknown	20	17	24	17	17	24	0.68

Data from the two study villages (Mélapleu and Zouatta II) in western Côte d'Ivoire were pooled.

a All values are percentages.

### Perception, treatment, and prevention of intestinal schistosomiasis

Table 5 summarizes the frequencies of symptoms, causes, treatment, and prevention of intestinal schistosomiasis among respondents, stratified by village. Less than half of the respondents (43%) claimed to have knowledge of intestinal schistosomiasis; significantly more in Zouatta II (75%) than in Mélapleu (14%). In Zouatta II, the main source of information pertaining to intestinal schistosomiasis was from health workers associated with the schistosomiasis research project (66% in Zouatta II *vs.* 25% in Mélapleu; χ^2^ = 9.4, *P* = 0.002). In Mélapleu, schools were cited as the most important source of information (62%), with a statistically highly significant difference to Zouatta II (18%; χ^2^ = 13.4, *P*<0.001).

**Table 5:** Knowledge of signs and symptoms, perceived causes, treatment, and prevention of intestinal schistosomiasis in 2003/2004.

Variable	Total^a^	Village		χ^2^	*P*-value
		Zouatta IIa,b	Mélapleua,c		
Knowledge (n = 207)	43	75	14	77.31	<0.001
Signs and symptoms^d^ (n = 88)					
Blood in stool	93	97	75	10.17	<0.001
Blood in urine	86	89	75	2.14	0.143
Abdominal pain	81	85	63	4.14	0.042
Vomiting	75	75	75	0.00	1.000
Diarrhea	73	75	63	1.03	0.310
Dysentery	38	39	31	0.32	0.568
Perceived causes					
Drinking contaminated water	86	93	56	15.05	<0.001
Bathing in dirty water	83	88	63	5.78	0.016
Open defecation in water	71	74	56	1.89	0.169
Malnutrition	57	65	19	N/A	<0.001^e^
Consumption of dirty fruits	51	61	6.3	N/A	<0.001^e^
Inborn disease	13	14	0	N/A	N/A
Place of treatment					
Dispensary/hospital	88	86	94	0.69	0.403
Family	35	43	0	N/A	N/A
Traditional healer	15	18	0	N/A	N/A
Type of treatment					
Pharmaceutical medicine	85	85	88	0.08	0.777
Traditional medicine	35	42	0	N/A	N/A
Medicine sold on street markets	27	29	19	0.71	0.397
Prevention^f^ (n = 80)					
Avoiding drinking dirty water	89	93	60	9.64	0.002
Avoiding bathing in dirty water	86	86	90	0.12	0.724
Avoiding open defecation in water	74	72	80	0.29	0.587
Avoiding unripe fruit consumption	69	75	20	N/A	<0.001^e^

Data were compared between two villages (Mélapleu and Zouatta II) in western Côte d'Ivoire.

N/A, not applicable.

a All values are percentages.

b Village subjected to community-based research and control activities.

c Village subjected to school-based research and control activities.

d Only people who gave an affirmative response for knowledge of schistosomiasis were included in this analysis.

e *P*-value based on Fisher's exact test.

f Only people who gave an affirmative response for prevention were included in this analysis.

Blood in stool was the most common symptom associated with intestinal schistosomiasis; it was mentioned by 97% of respondents in Zouatta II. Among potential sources of infection, drinking dirty surface water was significantly more often reported in Zouatta II (93%) than in Mélapleu (56%). Additionally, bathing in dirty surface water was identified as a source of infection. Fourteen percent of respondents from Zouatta II perceived schistosomiasis as an inborn disease.

Qualitative data showed that schistosomiasis was commonly confused with bloody diarrhea, dysentery, or hemorrhoids, particularly among women. This is supported by the following quote from a woman in Mélapleu “*We don't know ‘bilharziose’, we think all this is dysentery*”. We chose “blood in stool” as a variable to reconstruct experiences concerning intestinal schistosomiasis. Most of the participants stated that they or a relative had already experienced problems related to blood in stool at some point in the past. A young woman from Zouatta II explained: “*My mother had blood in stool and abdominal pain. When she went to the hospital, the doctor said that she had ‘bilharziose’*”. However, this symptom was referred to as dysentery, hemorrhoids, or worms rather than to intestinal schistosomiasis. Dysentery was a familiar disease process in the studied communities and regularly appeared in discussions. Still, blood in stool was seen to be a serious health problem and was believed among villagers to be caused by a parasite that is destructive to the liver and concomitantly drinks the host's blood. Many villagers sought treatment because of these perceived damaging effects. Other villagers, however, failed to seek treatment because they did not know the symptoms of intestinal schistosomiasis. In FGDs, none of the women were able to give an accurate description of the signs of the disease. Participants from Zouatta II were aware of the risks involved with direct water contacts. According to the qualitative data from the FGDs, most believed that drinking dirty water is the main route of acquiring the disease. For example, “*We have no potable water here; we drink water from the well and open sources. All this gave us diseases like ‘bilharziose’*”, said a 25-year-old woman from Zouatta II. This perception, in turn, affected people's protective measures.

Table 6 shows the causes, treatment, and prevention of intestinal schistosomiasis among the respondents, stratified by educational attainment and age. There were no significant differences between the categories with regard to perceived causes of intestinal schistosomiasis. However, a difference was noted between illiterate individuals who cited traditional medicines and drugs obtained on local markets more often than participants with at least primary schooling.

**Table 6:** Perceived causes, treatment, and prevention of intestinal schistosomiasis among participants who previously heard about schistosomiasis in 2003/2004.

Variable	Total(n = 88)^a^	Educational attainment			χ^2^	*P-*value	Age group (years)			χ^2^	*P-*value
		Illiterate (n = 38)^a^	Primary school(n = 28) a	Secondary school and higher(n = 22)^a^			18–35(n = 28)^a^	36–60(n = 42)^a^	>60(n = 18)^a^		
Perceived causes											
Drinking dirty water	86	89	89	77	2.05	0.357	75	90	94	4.67	0.097
Bathing in dirty water	83	82	79	91	1.41	0.493	71	93	78	5.88	0.053
Open defecation in water	71	71	71	68	0.07	0.964	71	71	67	0.15	0.925
Malnutrition	57	63	50	55	1.19	0.549	54	67	39	4.13	0.126
Consumption of dirty fruits	51	55	46	50	0.51	0.772	43	55	56	1.12	0.568
Inborn disease	38	19	7.1	4.5		0.18^b^	0	12	28	N/A	0.015^b^
Place of treatment											
Dispensary/hospital	88	84	93	86	1.13	0.566	96	83	83	2.99	0.224
Family	35	50	25	23	6.42	0.04	18	40	50	5.93	0.052
Traditional healer	15	24	7.1	7.1	0.15	0.119	11	9.5	33	N/A	0.045^b^
Type of treatment											
Pharmaceutical medicine	85	84	96	72	5.55	0.062	86	81	94	1.82	0.401
Traditional medicine	35	55	25	14	N/A	0.002 b	18	40	50	5.93	0.052
Medicine sold on street markets	27	42	18	14	N/A	0.023 b	25	26	33	0.43	0.806
Prevention											
Avoiding drinking dirty water	89	97	92	71	0.01	0.010	83	90	94	1.46	0.481
Avoiding bathing in dirt water	86	86	80	95	2.28	0.319	96	83	76	3.14	0.208
Avoiding open defecation in water	68	69	80	71	0.99	0.609	74	80	53	4.62	0.099
Avoiding unripe fruit consumption	69	80	68	48	6.31	0.043	50	69	67	2.76	0.251

Data were pooled for the two study villages (Mélapleu and Zouatta II) and stratified by educational attainment and age groups.

N/A, not applicable.

a All values are percentages.

b *P*-value based on Fisher's exact test.

Qualitative data confirmed these findings in terms of prevention, illustrated by the following quote from a 42-year-old man from Mélapleu: “*If someone knows the way of the transmission of a disease, he can avoid it. But, we don't know how we contract ‘bilharziose’, therefore we cannot avoid it. I know ‘bilharziose’ from those who went to the hospital and the doctor told them that they had this disease.*”

Table 7 shows the knowledge, treatment, and prevention among study participants, stratified by wealth quintiles. The poorest quintile mentioned blood in stool as the main symptom of intestinal schistosomiasis and used traditional medicine more often than the least poor. For prevention, the poorest cited avoiding drinking dirty water more often than their wealthier counterparts who, in turn, indicated more frequently to avoid bathing in dirty water. The majority of the participants mentioned that they avoided water contact as a preventive measure.

**Table 7:** Knowledge of signs and symptoms, perceived causes, treatment, and prevention of schistosomiasis, stratified by wealth quintiles, in 2003/2004.

Variable	Total^a^	Wealth quintile					Ratio(most poor/least poor)
		Most poor^a^	Very poor^a^	Poor^a^	Less poor^a^	Least poor^a^	
Knowledge of schistosomiasis (n = 207)	43	23	20	20	15	22	1.03
Signs and symptoms (n = 88)							
Blood in stool	93	100	89	94	92	89	1.11
Blood in urine	86	90	83	83	85	89	1.00
Abdominal pain	81	80	83	78	77	84	0.95
Vomiting	75	90	78	67	62	74	1.22
Diarrhea	73	70	83	72	69	68	1.02
Dysentery	38	55	28	28	38	37	1.49
Perceived causes							
Drinking dirty water	86	90	83	89	92	79	1.14
Bathing in dirty water	83	90	83	83	77	79	1.14
Open defecation in water	71	90	67	78	38	68	1.31
Malnutrition	57	63	72	61	38	42	1.49
Consumption of dirty fruits	51	60	56	44	46	47	1.22
Place of treatment							
Dispensary/hospital	88	90	78	83	92	95	0.95
Family	35	40	50	39	13	26	1.34
Traditional healer	15	10	22	28	7.6	5.2	1.92
Type of treatment							
Pharmaceutical medicine	85	80	78	83	92	95	0.84
Traditional medicine	35	55	44	33	18	21	2.61
Drugs sold on street markets	27	25	22	39	23	26	0.90
Prevention^b^ (n = 80)							
Avoiding drinking dirty water	89	85	83	83	85	74	1.15
Avoiding bathing in dirty water	80	75	83	83	69	84	0.89
Avoiding open defecation in water	74	75	83	67	46	58	1.29
Avoiding unripe fruit consumption	69	65	78	50	77	47	1.37
Source of information							
Hospital	61	70	61	44	85	53	1.33
Health worker^c^	59	65	61	56	69	42	1.54
Radio	51	55	56	33	62	53	1.04
Television	48	45	50	44	38	58	0.77
Family	35	35	28	50	38	26	1.33
School	26	30	17	22	31	32	0.95
Traditional healer	11	10	0	17	7.6	16	0.63

Data from the two study villages (Mélapleu and Zouatta II) in western Côte d'Ivoire were pooled.

a All values are percentages.

b Only people who gave an affirmative response for prevention were included in this analysis.

c Schistosomiasis project staff.

### Water contact behavior

Table 8 summarizes the principal reasons for water contact. We observed that pumps supplying potable water in each community were broken, and hence access to clean drinking water was difficult. The most frequent domestic activities placing individuals in contact with contaminated water were bathing in rivers, washing clothes, and crossing rivers. In each village, significant differences were found for washing dishes, bathing children, fetching drinking water, farming, fishing, swimming, and playing (all *P*<0.05).

**Table 8:** Reasons for water contact among respondents, stratified by study village, in 2003/2004.

Reason for water contact	Total(n = 207)^a^	Village		χ^2^	*P-*value
		Zouatta IIa,b(n = 96)	Mélapleua,c (n = 111)		
Bathing	92	89	96	3.49	0.062
Washing clothes	91	93	90	0.44	0.505
Crossing rivers	65	72	59	3.49	0.061
Washing dishes	65	51	77	14.7	<0.001
Washing children	65	44	82	34.5	<0.001
Fetching water	56	43	67	12.0	0.001
Fishing	53	62	45	6.29	0.012
Swimming	38	51	27	12.6	<0.001
Farming	21	14	28	6.36	0.012
Playing	6.3	10	3.0	5.20	0.023
Religious practices	3.9	3.0	5.0	0.26	0.608

a All values are percentages.

b Village subjected to community-based research and control activities.

c Village subjected to school-based research and control activities.

Data from non-participant observation revealed that water contact patterns were similar in both villages. Rivers are frequently used for both occupational and recreational activities. Some women mentioned that they preferred to wash clothes in rivers because suitable washing stones were available and there was time for social interaction and community chores. We observed water contact behavior in both villages that could lead to contamination, such as women washing clothes, fishing, and children bathing in the river simultaneously. Moreover, we found evidence of open defecation along riverbanks.

## Discussion

The aim of this study was to deepen our understanding and compare the local KAP of parasitic worm infections in two rural communities of western Côte d'Ivoire that were subjected either to school-based (Mélapleu) or community-based research and control activities (Zouatta II) against intestinal schistosomiasis and soil-transmitted helminthiasis. Both qualitative and quantitative methods were employed and data were triangulated. Since heads of households are a strong and recognized component of the cultural and social setting [43], [44], special emphasis was placed on interviewing these individuals.

Demographic and socio-economic profiles in the two villages were similar with the exception of ethnicity, religion, and marital status. Interestingly, when household heads were asked about health problems within the household and the community in general, intestinal worms and schistosomiasis were rarely mentioned spontaneously as a health problem of prime importance. This observation does not suggest that these infections are negligible, since worms were mentioned to be a serious health problem upon further probing. Most of the household interviewees possess more detailed knowledge of how a worm infection occurs and what measures are available for prevention than for schistosomiasis. The term ‘bilharziose’ was more readily understood in Zouatta II than in Mélapleu, although there was only little knowledge on the mode of transmission and how to prevent schistosomiasis, even in Zouatta II.

Our analysis of people's representations and practices against worms revealed that knowledge and practices lacked clear linear relationships. The following two considerations are offered for discussion. First, schistosomiasis and soil-transmitted helminthiasis are deeply engrained in social-ecological systems [45], and hence the way of life and common behaviors are difficult to change. For example, open defecation was said to be more comfortable than in latrines. With regard to water supply, the water from unprotected open sources was reported to be of better taste compared to that from wells. Second, there was a lack of alternatives for the clean drinking water and improved sanitation. For example, the pumps which would have provided safe drinking water were not functional in the two villages at the time of the survey, and only one out of four households surveyed had latrines.

Previous studies carried out in other schistosome-endemic areas found a comparatively higher level of awareness of schistosomiasis than was observed in these two villages of western Côte d'Ivoire. For example, Ndamba (1998) reported that 80% of villagers in Zimbabwe were aware of schistosomiasis [46]. In a community in south-eastern Nigeria, 42% of respondents had a clear perception of the disease and 27% were aware of high prevalence rates [47]. Prior research conducted in Brazil [22] and Egypt [48] revealed that people were fairly familiar with schistosomiasis. It is conceivable that the better knowledge of intestinal schistosomiasis in Zouatta II than in Mélapleu is attributable to the health information conveyed through a community-based approach. Indeed, respondents from Zouatta II mentioned that research and control activities served as their main source of information. Moreover, some villagers noted that information on schistosomiasis was passed on to them through family members, thus indicating that community social organizations may act as an intermediary to deliver health education messages. A Senegalese study [49] demonstrated lower awareness of intestinal schistosomiasis among the population despite several years of health education, using a diversity of communicational outlets, including radio, television, and posters, among others. Lessons learned in Senegal point out that a research project can have a positive effect on raising local knowledge, and it was concluded that intensive community-based actions are an effective means to do so. As participants from Mélapleu said that they learned about disease transmission at school, including and further strengthening the role of teachers in health education and community health promotion should be envisaged. Both the school and community structures should be integrated in such a health education program, so that school children can act as agents for the diffusion of health education messages. Indeed, studies from Brazil indicate that schools are a key source of information for schistosomiasis [22].

Passive case detection may be considered as a potential control measure if drugs are available at local and regional health facilities. An important mechanism to enhance health outcomes is to teach local community members (e.g., community health workers and teachers) about the signs and symptoms of worm infection, and implementing a community outreach treatment and prevention program. With this type of control strategy, however, community perceptions of diseases are particularly important in ensuring the control strategy's effectiveness, as perception will affect compliance [50]. In fact, recent evidence suggests that in the north-western part of Uganda communities are increasingly resisting to regularly take anthelmintic drugs as part of preventive chemotherapy programs targeting multiple neglected tropical diseases simultaneously [51]. In our study, treatment by passive case detection should be considered as most of the respondents trusted pharmaceutical drugs. However, people used both modern and traditional medicine to treat worm infections; the majority of them considered modern medicine to be inaccessible, and used traditional medicine or drugs sold on local markets. This is consistent with findings from a study in Egypt, where it was found that traditional medications were frequently used, since modern treatments were either unavailable due to high costs or lack of supply [48].

With regard to the transmission of parasitic worms, a common belief in our study villages was that this primarily was through food. In particular, consumption of meat, sweets, and over-ripe fruits were perceived as key factors in disease transmission. The relationship between consumption of specific foodstuffs and intestinal helminth infections likely relates to the associated abdominal symptoms. For example, consumption of spoiled meat or over-ripe fruits is thought to cause symptoms of bloating and cramping, similar to helmintic infections. Curtale and colleagues [52] made similar observations in Egypt, where a close association between food and helminth infection was established. The perception relating food with helmintic disease affected the behavior of the current study populations regarding protective measures. In many instances, children with helminth infections were not allowed to consume either meat or fruit. From a public health perspective, this is an important issue as these food items are of high nutritional value. Hence, restricting certain foodstuffs as they are perceived to be associated with helmintic infection may lead to, or further exacerbate, delayed child development [21], [53], [54]. Furthermore, our quantitative results revealed that soil was an unknown source of transmission for intestinal worm infections. This was confirmed during FGDs; only one woman in a FGD mentioned that infection could be transmitted when children eat soil. With regard to intestinal schistosomiasis, we did not identify any particular beliefs within the communities. This finding is in contrast to urinary schistosomiasis; common beliefs such as hematuria have been reported as a sign of puberty for male individuals [55].

Nonetheless, the observed low awareness of intestinal schistosomiasis in an area of high endemicity is intriguing, and clearly demonstrates the difficulty in comparing the biomedical and popular conception of disease. In fact, intestinal schistosomiasis is frequently confused with other diseases that have the same or similar symptoms. In general, there seems to be a lack of consistency between the transmission and preventive measures taken against schistosomiasis and other parasitic worm infections. Commonly, people's knowledge is a combination of endogenous and exogenous wisdom. Indeed, respondents gave answers in both categories; there was knowledge with a clear biomedical root obtained from health workers and the research team on one hand, and information obtained from the cultural milieu on the other hand. For example, people mentioned that wearing shoes is a preventive measure against worms, without mentioning that soil contact is necessary for worm transmission. The knowledge of shoes preventing soil-transmitted helminth infections was obtained or reinforced by the research team during the meetings preceding the 2004 survey. It follows that knowledge of the modes of transmission and prevention of worm infections was moderate at best. Improving the public's perception of schistosomiasis would require sustained control efforts and intensive educational actions. Yet, enhanced knowledge and practices do not necessarily lead to behavioral changes. For example, even if the public's awareness about infection risks improves, people will continue to visit contaminated water sources if there are no readily available alternatives, the fetching of water at open water sources continues to be related to important social interaction, and the taste of current water sources is perceived as better than the water obtained from wells [56]–[58]. Hopefully following a community-based health education campaign, knowledge of common symptoms and transmission may lead to improved health-seeking behavior and early treatment [59], [60].

We found two health-conscience behaviors, namely hand washing before eating and hand washing after defecation. While these behaviors are laudable, they are unfortunately overshadowed by other practices of poor hygiene. For example, only 25% of households had a latrine and despite this, villagers tend to defecate where convenient – still rarely using latrines where available. Such practices are dangerous as the number of helminth eggs produced by one adult female worm is so large that a single contaminated stool passed in the soil is sufficient to infect an entire village [61]. It would be interesting to try a community-led total sanitation approach (CLTS) and measure the impact of such a strategy on re-infection patterns of soil-transmitted helminthiasis, intestinal schistosomiasis and other neglected tropical diseases [17]. CLTS is an integrated approach to achieve and sustain an open defecation-free status of communities through a participatory approach. It facilitates the critical analysis by the community of their own sanitation profile, their practices of defecation and the consequences, leading to collective action to become open defecation-free, and hence is accompanied by environmental measures to improve sanitation, hygiene practices, waste disposal, and protection of clean drinking water sources [62].

In conclusion, our results emphasize that current morbidity control through preventive chemotherapy primarily targeting school-aged children has limitations. Older population segments are insufficiently addressed, and hence new knowledge on prevention and control of parasitic worm infections is minimal. Improved access to clean water and sanitation is necessary for sustainable control of major helminthiases, and participatory approaches such as CLTS should be attempted. Our results further suggest that educational programs should target areas of high endemicity with community-based interventions. We recommend that household heads from all socio-economic segments should be specifically targeted, so that they can transmit important health information to their children and relatives. For such a program to be effective, a continuous surveillance process directed at monitoring passive case detection, assessing the impact of health education messages, and evaluating change in infection patterns are necessary.

## Supporting Information

Alternative Language Abstract S1 Translation of the abstract into French by Cinthia Acka
(.27 MB DOC)

Alternative Language Abstract S2 Translation of the abstract into German by Jürg Utzinger and Jennifer Keiser
(.28 MB DOC)

Alternative Language Abstract S3 Translation of the abstract into Italian by Giovanna Raso and Aurelio Di Pasquale
(.27 MB DOC)
