# Supplementary material for: Parasitic Worms: Knowledge, Attitudes, and Practices in Western Côte d’Ivoire with Implications for Integrated Control
Source: PLoS Negl Trop Dis. 2010 Dec 21;4(12):e910. doi: 10.1371/journal.pntd.0000910 (PMC3006135; doi:10.1371/journal.pntd.0000910)
Supplement: Alternative Language Abstract S1 — Translation of the abstract into French by Cinthia Acka (.27 MB DOC) [file pntd.0000910.s001.doc]

**Affections Parasitaires : Connaissances, Attitudes et Pratiques à l’Ouest de la Côte d’Ivoire et Implications pour une Lutte Intégrée**

**Résumé**

***Contexte :*** Dans les pays en voie de développement où les infections parasitaires sont omniprésentes, la chimiothérapie est la stratégie clé pour le contrôle de la morbidité. Cependant, les connaissances locales, les attitudes et les pratiques (CAP) liées à ces parasitoses sont très peu connues, bien que de telles informations soient nécessaires pour la prévention et le contrôle durable de ces affections.

***Méthodes :*** Nous avons conduit une étude CAP dans deux communautés rurales en Côte d’Ivoire dans lesquelles des activités de recherche et de lutte contre les parasitoses au niveau scolaire et au niveau communautaire ont été menées. Nous avons eu recours aux méthodes qualitatives et quantitatives. La première approche incluait des interviews approfondies avec des informateurs clés, des discussions de groupes focaux avec des élèves et des adultes. Les méthodes quantitatives ont consisté à l’administration d’un questionnaire standardisé aux chefs de ménage.

***Résultats Principaux :*** Les deux communautés soumises à l’étude étaient dépourvues d’eau potable et seulement le quart des ménages avait des latrines fonctionnelles. Il y avait une meilleure connaissance de la transmission des vers intestinaux par rapport à la schistosomiase intestinale, mais les interlocuteurs ayant bénéficié des interventions basées sur la communauté avaient une connaissance améliorée de la schistosomiase. Dans le village où il y a eu les interventions basées sur la communauté, trois quart des ménages interviewés connaissaient mieux la schistosomiase intestinale par rapport au 14% de ceux provenant du village où les interventions étaient focalisées sur l’école (*P<*0,001). Aussi, pendant que deux tiers des répondants provenant du village où il y a eu les interventions basées sur la communauté ont mentionné le projet de recherche et de lutte contre les parasitoses comme source principale d’information, seulement le quart des répondants du village avec interventions basées sur l’école a cité le projet comme source d’information relative à la schistosomiase intestinale.

***Conclusions/Implications :*** La prévention des parasitoses hydriques ciblant uniquement les enfants d’âge scolaire a des limites, notamment lorsque que les adultes sont négligés et que les connaissances relatives à la prévention et au contrôle de ces affections sont insuffisantes. L’amélioration de l’accès à l’eau potable combinée avec l’éducation pour la santé dans la lutte contre les infections parasitaires, a un impact plus durable.

***Traduction :*** Cinthia A. Acka
