# Supplementary material for: Parasitic Worms: Knowledge, Attitudes, and Practices in Western Côte d’Ivoire with Implications for Integrated Control
Source: PLoS Negl Trop Dis. 2010 Dec 21;4(12):e910. doi: 10.1371/journal.pntd.0000910 (PMC3006135; doi:10.1371/journal.pntd.0000910)
Supplement: Alternative Language Abstract S2 — Translation of the abstract into German by Jürg Utzinger and Jennifer Keiser (.28 MB DOC) [file pntd.0000910.s002.doc]

**Verständnis, Verhalten und lokale Praktiken betreffend parasitären Wurminfektionen im Westen der Elfenbeinküste und deren Bedeutung für integrierte Kontrollmassnahmen**

**Zusammenfassung**

***Hintergrund:*** Parasitäre Wurminfektionen sind vor allem in Entwicklungsländern weit verbreitet. Die wichtigste Strategie für die Kontrolle von parasitären Wurminfektionen beruht auf der regelmässigen Entwurmung von Hochrisikogruppen wie zum Beispiel Schulkindern. Allerdings fehlt es oft an einem fundierten Wissen über lokale Verhaltensmuster und Praktiken um Wurminfektionen zu vermeiden oder gezielt zu bekämpfen.

**Methoden:** Mittels einer Querschnittsstudie untersuchten wir das Verständnis, Verhalten und lokale Praktiken zur Prävention und Kontrolle von parasitären Wurminfektionen in zwei Dörfern der Elfenbeinküste, welche von Interventionen auf Schul- oder Dorfebene profitiert hatten. Daten wurden mittels qualitativen Methoden (z.B. direkte Beobachtungen, vertiefte Interviews und Gruppendiskussionen mit Schulkindern und älteren Bevölkerungsgruppen) und quantitativen Methoden (z.B. strukturierter Fragebogen welcher an alle Haushalte verteilt wurde) erhoben.

**Wichtigste Ergebnisse:** Zugang zu sauberem Trinkwasser fehlte in beiden Dörfern und lediglich ein Viertel der Haushalte hatte funktionierende Latrinen. Die Bevölkerung hatte ein besseres Verständnis von durch Bodenkontakt übertragenen Wurmerkrankungen im Vergleich zur Bilharziose. Interventionen auf Gemeindeebene resultierten in einem verbesserten Verständnis der Bilharziose. In der Tat wussten rund drei Viertel der Bevölkerung wo Forschung und Interventionen bezüglich parasitären Wurminfektionen auf Gemeindeebene durchgeführt wurden von der Bilharziose, während lediglich 14% über Interventionen auf Schulniveau informiert waren (*P*<0,001). Rund zwei Drittel der Bevölkerung des Dorfes wo Interventionen auf Gemeindeebene durchgeführt wurden, gaben an, dass unser Forschungs- und Kontrollprogramm die Hauptquelle des Wissens war. Als Vergleich gaben lediglich ein Viertel der Bevölkerung wo Schulinterventionen durchgeführt wurden an, unser Projekt diente als Hauptquelle dieser Information.

***Schlussfolgerungen/Bedeutung:*** Regelmässige Entwurmungskampagnen von Schulkindern haben Schwächen, da ältere Bevölkerungsgruppen oft zuwenig berücksichtigt werden. Daher fehlt dieser Altersgruppe oft ein fundiertes Wissen über die Prävention und Kontrolle von parasitären Wurminfektionen. Ein verbesserter Zugang zu sauberem Trinkwasser und sanitären Einrichtungen, gekoppelt mit Gesundheitsausbildung sind wichtig, um die nachhaltige Kontrolle von Wurminfektionen voranzutreiben.

***Übersetzung:*** Jürg Utzinger & Jennifer Keiser
