# Supplementary material for: Parasitic Worms: Knowledge, Attitudes, and Practices in Western Côte d’Ivoire with Implications for Integrated Control
Source: PLoS Negl Trop Dis. 2010 Dec 21;4(12):e910. doi: 10.1371/journal.pntd.0000910 (PMC3006135; doi:10.1371/journal.pntd.0000910)
Supplement: Alternative Language Abstract S3 — Translation of the abstract into Italian by Giovanna Raso and Aurelio Di Pasquale (.27 MB DOC) [file pntd.0000910.s003.doc]

**Vermi Parassiti: Conoscenza, Atteggiamenti, e Prassi nella Costa d’Avorio Occidentale con Implicazioni per un Controllo Integrato**

***Background:*** Nei paesi in via di sviluppo in cui le infezioni parassitarie sono dilaganti, la prevenzione attraverso la chemioterapia è la strategia chiave per il controllo della morbilità. Tuttavia, le conoscenze, atteggiamenti e prassi locali associate a queste infezioni sono scarsamente comprese, sebbene tali informazioni sono necessarie per la prevenzione e il controllo sostenibile di queste malattie.

***Metodi:*** Abbiamo condotto uno studio in due comunità rurali in Costa d'Avorio sulle conoscenze, atteggiamenti e prassi locali con attività di ricerca e controllo sia a livello scolastico che comunitario. Sono stati usati metodi sia qualitativi che quantitativi. Il primo approccio riguarda in particolare, interviste approfondite con “informatori chiave”, e discussioni di gruppo mirate con bambini in età scolare ed adulti. Come metodo quantitativo è stato utilizzato un questionario strutturato sottoposto ai capi famiglia.

***Principali Risultati:*** Le due comunità oggetto di studio non avevano accesso all’acqua potabile e solo un quarto delle famiglie disponeva di servizi igienici funzionanti. Nonostante la maggior comprensione della trasmissione di geoelmintiasi rispetto alla schistosomiasi intestinale, gli interventi condotti a livello della comunità piuttosto che quelli condotti a livello scolastico hanno migliorato la conoscenza della schistosomiasi. Nel villaggio in cui gli interventi sono stati effettuati a livello comunitario, tre quarti delle famiglie intervistate avevano una maggiore conoscenza della schistosomiasi intestinale rispetto al 14% delle famiglie del villaggio dove gli interventi sono stati effettuati a livello scolastico (*P* <0,001). Mentre nel villaggio dove sono stati effettuati gli interventi a livello comunitario due terzi degli intervistati hanno segnalato di avere ottenuto le informazioni sulla schistosomiasi intestinale grazie al progetto di ricerca e di controllo delle malattie parassitarie, nel villaggio dove gli interventi sono stati effettuati a livello scolastico solo un quarto degli intervistati hanno citato il progetto di ricerca e controllo come fonte d’informazione.

***Conclusioni/Implicazioni:*** La prevenzione delle infezioni parassitarie solo a livello scolastico ha alcune limitazioni, dato che il segmento della popolazione adulta viene trascurato e quindi vi e’ una mancanza di conoscenza su come prevenire e controllare le infezioni parassitarie. E’ necessario migliore accesso all'acqua potabile e ai servizi igienici, insieme a un l'educazione alla salute per ottenere un impatto duraturo nella lotta contro le geoelmintiasi.

*Traduzione:* Giovanna Raso & Aurelio Di Pasquale
